# A phase Ib multiple ascending dose study evaluating safety, pharmacokinetics, and early clinical response of brodalumab, a human anti-IL-17R antibody, in methotrexate-resistant rheumatoid arthritis

**DOI:** 10.1186/ar4347

**Published:** 2013-10-25

**Authors:** David A Martin, Melvin Churchill, Luis Felipe Flores-Suarez, Mario H Cardiel, Daniel Wallace, Richard Martin, Kristine Phillips, Jeffrey L Kaine, Hua Dong, David Salinger, Erin Stevens, Chris B Russell, James B Chung

**Affiliations:** 1 Amgen Inc, 1201 Amgen Court West, Seattle, WA 98119, USA; 2 Arthritis Center of Nebraska, 3901 Pine Lake Road, Lincoln, NE 68516, USA; 3 Primary Systemic Vasculitis Clinic, National Institute of Respiratory Diseases, México City, Mexico; 4 Clinical Research Center of Morelia, Morelia, Mexico; 5 Division of Rheumatology, Cedars-Sinai Medical Center, Los Angeles, CA, USA; 6 Division of Rheumatology, College of Medicine, Michigan State University, 775 Ball Avenue NE, Grand Rapids, MI 49503, USA; 7 Scleroderma Program, Division of Rheumatology, School of Medicine, University of Michigan, 7C27 NIB, 300 N Ingalls Street, Ann Arbor, MI 48109, USA; 8 Sarasota Arthritis Center, 3500 S Tamiami Trail, Sarasota, FL 34239, USA; 9 Amgen Inc, One Amgen Center Drive, Thousand Oaks, CA 91320, USA; 10 Present address: Gilead Sciences, 333 Lakeside Drive, Foster City, CA 94404, USA

## Abstract

**Introduction:**

The aim of this study was to evaluate the safety, pharmacokinetics, and clinical response of brodalumab (AMG 827), a human, anti-IL-17 receptor A (IL-17RA) monoclonal antibody in subjects with moderate-to-severe rheumatoid arthritis (RA).

**Methods:**

This phase Ib, randomized, placebo-controlled, double-blind multiple ascending dose study enrolled subjects with moderate to severe RA (≥6/66 swollen and ≥8/68 tender joints). Subjects were randomized 3:1 to receive brodalumab (50 mg, 140 mg, or 210 mg subcutaneously every two weeks for 6 doses per group; or 420 mg or 700 mg intravenously every 4 weeks for two doses per group) or placebo. Endpoints included incidence of adverse events (AEs) and pharmacokinetics. Exploratory endpoints included pharmacodynamics, and improvements in RA clinical metrics.

**Results:**

Forty subjects were randomized to investigational product; one subject discontinued due to worsening of RA (placebo). The study was not designed to assess efficacy. AEs were reported by 70% (7/10) of placebo subjects and 77% (22/30) of brodalumab subjects. Three serious AEs were reported in two subjects; there were no opportunistic infections. Brodalumab treatment resulted in inhibition of IL-17 receptor signaling and receptor occupancy on circulating leukocytes. No treatment effects were observed with individual measures of RA disease activity. On day 85 (week 13) 37% (11/30) of brodalumab subjects and 22% (2/9) of placebo subjects achieved ACR20; 7% (2/30) brodalumab subjects and 11% (1/9) of placebo subjects achieved ACR50; and 0% (0/30) brodalumab subjects and 0% (0/9) of placebo subjects achieved ACR70.

**Conclusions:**

Multiple dose administration of brodalumab was tolerated in subjects with active RA. There was no evidence of a clinical response to brodalumab in subjects with RA.

**Trial registration:**

ClinicalTrials.gov, NCT00771030

## Introduction

Rheumatoid arthritis (RA) is an autoimmune disease which produces synovitis in diarthrodial joints and is characterized by the presence of autoreactive T and B cells and the production of proinflammatory cytokines, leading to cartilage and bone damage [1]. RA occurs in approximately 1% of adults of all races worldwide [1]. Conventional treatment with disease-modifying antirheumatic drugs (DMARDs) has been augmented by the introduction of targeted biologics that specifically inhibit proinflammatory cytokines [2,3]. Despite the ability of these therapies to effectively suppress disease activity, only a minority of patients achieve adequate disease control (American College of Rheumatology 50% improvement criteria (ACR50)) or disease remission (Disease Activity Score in 28 joints (DAS28) less than 2.6) [1]. Thus there remains an unmet need for patients with RA that warrants the development of drugs for treatment that target new mechanisms of action.

T helper 17 (Th17) cells, a subset of CD4+ effector T helper cells distinct from the classic Th1 and Th2 lineages, provide innate and adaptive immunity against pathogens by orchestrating inflammation signaling through the induction of cytokine, chemokine and matrix metalloprotease expression [4]. The downstream effects of Th17 cells are driven by production of the interleukin 17 (IL-17) family of cytokines, most notably IL-17A and IL-17 F [5]. Aberrant Th17 responses and IL-17 production have been implicated in a variety of human autoimmune diseases, including psoriasis, RA, psoriatic arthritis (PsA) and multiple sclerosis [6,7].

The role of IL-17 in the pathogenesis of RA has been studied in both preclinical and clinical studies [5]. Increased levels of IL-17A have been detected in the synovial fluid of patients with RA [8-10], and blockade of IL-17A signaling can inhibit osteoclast formation induced by conditioned culture media of RA synovial tissues. Furthermore, children with juvenile inflammatory arthritis (JIA) have elevated IL-17A-positive T cells in inflamed joints [11]. In an *ex vivo* model using explanted synovial tissue from human RA patients, blockade of IL-17A reduced the spontaneous production of IL-6 and collagen breakdown products [12]. Results from early-phase studies of RA suggest clinical benefits of IL-17A blockade in RA patients [13,14].

Brodalumab is a human immunoglobulin G2 (IgG2) monoclonal antibody that binds with high affinity to human IL-17 receptor A (IL-17RA) and blocks the biological activity of IL-17A, IL-17 F and IL17-A/F heterodimer signaling through the IL-17RA/RC complex, as well as IL-25 (IL-17E) signaling through the IL-17RA/RB complex. Brodalumab has been shown to be capable of inhibiting the inflammatory process in psoriasis [7]. In this phase Ib, randomized, placebo-controlled, double-blind, multiple ascending dose study, we evaluated the safety, pharmacokinetics and early clinical response of brodalumab in patients with moderate to severe RA.

## Methods

### Participants

Men and women ages 18 to 70 years were eligible for participation in the study if they had had active RA as defined by American College of Rheumatology (ACR) criteria [15] for at least 6 months prior to screening, despite treatment with methotrexate (MTX) consecutively for 12 weeks or longer. Active RA was defined as six or more swollen joints; eight or more tender and/or painful joints; and any one or more of the characteristics erythrocyte sedimentation rate (ESR) 28 mm/h or higher, C-reactive protein (CRP) above 15 mg/L or morning stiffness for more than 45 minutes. Patients were required to be on a stable dose of oral or subcutaneous MTX (15 to 25 mg weekly) for at least 4 weeks. Patients currently taking nonsteroidal anti-inflammatory drugs (NSAIDs) or oral corticosteroids (not to exceed the equivalent of 10 mg/day prednisone) were required to be on a stable dose more than 4 weeks prior to screening and to remain on that stable dose during the treatment period of the study.

Patients who were given any commercial biologic DMARD within the 3 months prior to enrollment or rituximab within the 12 months prior to enrollment were excluded from participation in the study. Patients with evidence of any active, recurrent or recent infections or a history of malignancy or other autoimmune or uncontrolled systemic disease were also excluded. Patients with class IV RA [15], Felty’s syndrome or chronic pain syndrome requiring daily narcotic analgesics were excluded as well.

This study was approved by the medical ethics committee of all participating institutions and was performed according to the Declaration of Helsinki. All participants provided written informed consent prior to study enrollment.

### Study design

This study was a multicenter, randomized, double-blind, placebo-controlled, multiple ascending dose study in patients with RA conducted at 11 sites: 7 in the United States, 2 in Canada and 2 in Mexico. Patients were randomized 3:1 to receive ascending doses of brodalumab or placebo subcutaneously (cohorts 1 to 3) or intravenously (cohorts 5 and 6). Patients in cohorts 1 to 3 received brodalumab (50, 140 or 210 mg) subcutaneously every 2 weeks for a total of six doses, and those in cohorts 5 and 6 received brodalumab (420 or 700 mg) by intravenous (IV) infusion every 4 weeks for a total of two doses. Cohort 4 was designed to be used in a dose expansion study to provide evidence of biological impact in 70 patients with RA receiving a dose of brodalumab defined during the dose escalation phase of the study. This cohort was not enrolled, because experimental endpoints would be achieved during a separate phase II study. The dose levels of brodalumab administered in the study were determined on the basis of the results of a previous clinical study [7].

Dose escalations required acceptable safety data based on blinded review of safety data at a dose-level review meeting following completion of the day 15/week 3 visit by the final patient in each cohort and when six or more patients in a cohort had been administered at least three doses of brodalumab (cohorts 1, 2, 3 and 5). In cohort 6, dose escalation followed completion of the day 15/week 3 visit by the final patient in cohort 5 and six or more patients in cohort 5 had been administered two or more IV infusions of brodalumab. All study personnel remained blinded to treatment until study completion.

### Safety and clinical assessments

Safety was assessed by monitoring adverse events (AEs), serious adverse events (SAEs) and routine hematologic and laboratory values for all patients who received at least one dose of the investigational product (*n* = 40). RA disease assessments were completed during a screening visit and on day −1, 15, 29, 57 and 85 post–brodalumab administration. RA disease activity was assessed by scoring the number of swollen joints (*n* = 66) and tender joints (*n* = 68), by Disease Activity Score in 28 joints (DAS28) and by physician assessment of disease activity. All joint assessments were performed by an experienced, independent, blinded joint evaluator. The evaluator could not be the treating physician and could not interact with the patient throughout the study beyond the assessment of joints or for any other patient assessments or laboratory measures of efficacy. In addition to the DAS28, each patient completed a set of RA disease assessment questionnaires. These patient questionnaires included patient assessment of disease activity, patient assessment of pain, patient assessment of physical function and patient assessment of morning stiffness.

### Pharmacokinetics

Serum brodalumab concentration was quantified with a validated ELISA using a mouse anti-brodalumab monoclonal capture antibody (internal clone; Amgen, Thousand Oaks, CA, USA) and a horseradish peroxidase–conjugated mouse anti-brodalumab monoclonal detection antibody (internal clone; Amgen). The ELISA was developed with tetramethylbenzidine peroxide substrate solution (BioFX Laboratories, Owings Mills, MD, USA), and optical density signal was measured at 450 nm.

### Pharmacodynamics

IL-17 receptor occupancy (RO) was measured as relative brodalumab-bound receptors to total available receptors using flow cytometry (FACSCalibur; BD Biosciences, San Jose, CA, USA). Whole blood was stained with CD45 allophycocyanin and CD4 peridinin chlorophyll in combination with either a 1:1 brodalumab:phycoerythrin (PE) conjugate to report total available receptor, a 1:1 PE anti-mouse IL-17RA antibody (M204, internal clone; Amgen) conjugate to report total receptor expression level, or a human anti-keyhole limpet hemocyanin/anti-PE isotype control antibody. Heparinized blood samples were collected, and RO values were determined.

Functional baseline IL-17R signaling in the presence and absence of drug was examined by measuring IL-6 mRNA induction following a 4-hour exposure to TNF and a dose titration of IL-17. The IL-6 transcript was measured using a branched DNA signal amplification assay of cell lysates from whole blood collected prior to drug administration. The magnitude of IL-6 mRNA inductions and change in half-maximal effective concentration (EC_50_) values were calculated for brodalumab blockade in comparison to predose values.

### Immunogenicity assays

Serum samples were tested in an internally validated immunoassay (Amgen analytical method) for the presence of anti-brodalumab-binding antibodies. Equal concentrations of biotinylated and ruthenylated brodalumab in 1 M Tris, pH 9.5, were added to acid-treated patient serum samples. Following incubation at ambient temperature, samples were transferred to an avidin-coated MSD high bind plate (Meso Scale Discovery, Rockville, MD, USA). Plate wells were washed, MSD read buffer containing tripropylamine was added and the plate was read on the SECTOR Imager 6000 plate reader (Meso Scale Discovery). Ruthenylated brodalumab produced enhanced chemiluminescence signals proportionate to the concentration of anti-brodalumab antibodies in the sample. Samples found to be positive for brodalumab-binding antibodies were further tested using an internally validated, cell-based bioassay to determine if the antibodies were able to neutralize the activity of brodalumab. Human foreskin fibroblast cells were cultured in the presence of 5% patient serum and 75 ng/ml brodalumab for 2 hours prior to overnight incubation with 10 ng/ml recombinant human IL-17, which induces secretion of IL-8. The DELFIA TRF (dissociation-enhanced lanthanide fluorescent immunoassay with time-resolved fluorescence) assay platform (PerkinElmer, Waltham, MA, USA) was used to quantify secreted IL-8.

### Statistical methods

Demographic, safety, pharmacokinetic, pharmacodynamic and biomarker data were summarized descriptively by dose cohort. Descriptive statistics on continuous data included means, medians, standard deviations and ranges, and categorical data were summarized using frequency counts and percentages. As the primary endpoint for this study was safety/tolerability, no statistical powering was performed and the sample size was based on feasibility and historical approaches used for similar studies in this population. Analyses of safety endpoints were performed on all patients who were randomized and received at least one dose of the investigational product.

## Results

### Patient characteristics

All 40 patients enrolled in the study received at least one dose of investigational product, and 39 completed the study. One patient received IV placebo and was discontinued from study participation on day 85 because of increased disease activity that warranted systemic intervention. The demographics and baseline characteristics were generally similar among cohorts (Table 1). The majority of patients were women (85%) and white (45%) or Latino (48%). Their mean age was 51.4 years. Disease activity at baseline, including swollen and tender joints, patient and physician global assessments of disease activity and measures of systemic inflammation (ESR and CRP) were generally similar across the cohorts (Table 1).

**Table 1:** **Patient demographics and clinical characteristics**^**a**^

**Demographics and characteristics**				**Brodalumab**						
	**Placebo**			**SC**			**IV**			
	**SC**	**IV**	**All**	**50 mg**	**140 mg**	**210 mg**	**420 mg**	**700 mg**	**All**	**Total**
	**(*N*** **= 6)**	**(*N*** **= 4)**	**(*N *** **=10)**	**(*N*** **= 6)**	**(*N*** **= 6)**	**(*N*** **= 6)**	**(*N*** **= 6)**	**(*N*** **= 6)**	**(*N*** **= 30)**	**(*N*** **= 40)**
Females, *n* (%)	5 (83)	4 (100)	9 (90)	6 (100)	6 (100)	5 (83)	5 (83)	3 (50)	25 (83)	34 (85)
Race, *n* (%)										
White	4 (67)	2 (50)	6 (60)	3 (50)	2 (33)	1 (17)	5 (83)	1 (17)	12 (40)	18 (45)
Black	1 (17)	1 (25)	2 (20)	0 (0)	0 (0)	0 (0)	0 (0)	0 (0)	0 (0)	2 (5)
Latino	1 (17)	1 (25)	2 (20)	3 (50)	4 (67)	4 (67)	1 (17)	5 (83)	17 (57)	19 (48)
Asian	0 (0)	0 (0)	0 (0)	0 (0)	0 (0)	1 (17)	0 (0)	0 (0)	1 (3)	1 (3)
Age (yr)	52 (10)	56 (12)	53 (10)	46 (12)	57 (9)	46 (10)	56 (7)	50 (6)	51 (10)	51 (10)
Height (cm)	162 (8)	163 (5)	163 (7)	159 (13)	158 (8)	158 (13)	168 (6)	161 (10)	161 (11)	161 (10)
Weight (kg)	75 (10)	101 (28)	85 (22)	80 (17)	77 (12)	64 (11)	81 (12)	79 (14)	76 (14)	79 (17)
BMI (kg/m^2^)	29 (4)	37 (9)	32 (7)	33 (14)	31 (3)	26 (3)	29 (4)	31 (4)	30 (7)	30 (7)
Tender/painful joint counts	24 (12)	30 (21)	26 (15)	23 (12)	40 (26)	32 (27)	42 (21)	32 (12)	34 (21)	32 (20)
Swollen joint counts	15 (15)	19 (8)	17 (12)	10 (6)	22 (13)	20 (7)	19 (11)	21 (16)	18 (11)	18 (11)
Patient global assessment of disease activity	72 (23)	57 (22)	67 (23)	57 (16)	68 (23)	51 (25)	44 (18)	54 (23)	55 (21)	57 (22)
Physician global assessment of disease activity	7.7 (1.8)	6.0 (1.8)	7.0 (1.9)	6.7 (2.1)	6.3 (2.2)	7.3 (2.0)	6.8 (1.9)	7.0 (0.9)	6.8 (1.8)	6.9 (1.8)
Patient global assessment of pain	72 (23)	63 (18)	68 (20)	60 (18)	71 (17)	49 (28)	49 (14)	60 (24)	58 (21)	60 (21)
HAQ-DI	1.4 (0.9)	1.4 (0.5)	1.4 (0.7)	1.3 (0.7)	1.7 (0.6)	1.7 (1.0)	1.5 (0.7)	1.7 (0.5)	1.6 (0.7)	1.5 (0.7)
CRP (mg/L)	15 (17)	26 (39)	19 (26)	7 (9)	7 (4)	28 (39)	24 (50)	33 (53)	20 (36)	20 (34)
ESR (mm/h)	27 (23)	39 (29)	32 (26)	19 (11)	16 (11)	23 (15)	23 (28)	41 (45)	24 (26)	26 (25)
RF-positive, *n* (%)	4 (67)	4 (100)	8 (80)	5 (83)	4 (67)	5 (83)	5 (83)	5 (83)	24 (80)	32 (80)

^a^BMI, body mass index; CRP, C-reactive protein; ESR, erythrocyte sedimentation rate; HAQ-DI, Health Assessment Questionnaire Disability Index; IV, intravenous; RF, rheumatoid factor; SC, subcutaneous. All data are means (SD) unless otherwise indicated.

### Safety

AEs were reported by seven (70%) of ten patients administered placebo and by twenty-two (77%) of thirty patients administered brodalumab (Table 2). The most common AEs were headache (30%) and cough (13%), which both occurred more frequently in the placebo group (40% vs 27% and 20% vs 10%, respectively) than in the active treatment group.

**Table 2:** **Adverse events**^**a**^

				**Brodalumab**						
	**Placebo**			**SC**			**IV**			
**Adverse events**	**SC**	**IV**	**All**	**50 mg**	**140 mg**	**210 mg**	**420 mg**	**700 mg**	**All**	**Total**
	**(*N*** **= 6)**	**(*N*** **= 4)**	**(*N *** **=10)**	**(*N*** **= 6)**	**(*N*** **= 6)**	**(*N*** **= 6)**	**(*N*** **= 6)**	**(*N*** **= 6)**	**(*N*** **= 30)**	**(*N*** **= 40)**
Adverse events, *n* (%)										
Any	3 (50)	4 (100)	7 (70)	5 (83)	5 (83)	4 (67)	5 (83)	4 (67)	23 (77)	30 (75)
Serious^b^	0 (0)	1 (25)	1 (10)	0 (0)	0 (0)	0 (0)	1 (17)	0 (0)	1 (4)	2 (5)
Fatal	0 (0)	0 (0)	0 (0)	0 (0)	0 (0)	0 (0)	0 (0)	0 (0)	0 (0)	0 (0)
Leading to study discontinuation	0 (0)	1 (25)	1 (10)	0 (0)	0 (0)	0 (0)	0 (0)	0 (0)	0 (0)	1 (3)
Leading to IP discontinuation	0 (0)	0 (0)	0 (0)	0 (0)	0 (0)	0 (0)	0 (0)	1 (17)	0 (0)	0 (0)
Common adverse events^c^										
Headache	2 (33)	2 (50)	4 (40)	1 (17)	1 (17)	2 (33)	2 (33)	2 (33)	8 (27)	12 (30)
Cough	2 (33)	0 (0)	2 (20)	1 (17)	0 (0)	0 (0)	1 (17)	0 (0)	3 (10)	5 (13)
Abdominal pain	0 (0)	0 (0)	0 (0)	0 (0)	2 (33)	1 (17)	1 (17)	1 (17)	4 (13)	4 (10)
Constipation	1 (17)	1 (25)	2 (20)	0 (0)	0 (0)	2 (33)	0 (0)	0 (0)	2 (7)	4 (10)
Diarrhea	0 (0)	0 (0)	0 (0)	0 (0)	2 (33)	2 (33)	0 (0)	0 (0)	4 (13)	4 (10)
Upper respiratory tract infection	0 (0)	1 (25)	1 (10)	1 (17)	1 (17)	0 (0)	0 (0)	1 (17)	3 (10)	4 (10)
Rash	0 (0)	1 (0)	1 (10)	0 (0)	2 (33)	0 (0)	0 (0)	0 (0)	2 (7)	3 (8)

^a^IP investigational product; IV, intravenous; SC subcutaneous. ^b^A *serious adverse event* was defined as an event that was fatal or life-threatening, either required or prolonged hospitalization or caused persistent or substantial disability or incapacity or congenital anomaly or birth defect or an event that was considered by the investigator to be a medically important event. ^c^Common adverse events were those that were reported in at least three patients from all brodalumab-treated groups combined.

During the study, three SAEs were reported: complicated migraine in one patient (IV placebo group) and gastroesophageal reflux disease and noncardiac chest pain in another patient (in the brodalumab 420 mg IV group). None of the SAEs were considered by the investigator to be related to the investigational product. The investigational product was discontinued in one patient in the 700 mg IV brodalumab group following the first of two planned doses because of an AE of oropharyngeal candidiasis that was considered by the investigator not to be drug-related. One patient in the IV placebo group discontinued the study early because of an RA flare.

The frequency of treatment-related infections was low (7.5%), with no treatment-related infections reported in more than one patient, one case of cellulitis (placebo), one case of herpes zoster (brodalumab 420 mg IV group) and one upper respiratory tract infection (brodalumab 140 mg SC group). No deaths occurred during the study. One patient (brodalumab 50 mg SC) experienced transient decreased neutrophil counts (nadir 1.47 × 10^3^/μl on day 106), which normalized spontaneously by day 127 without an interruption of or decreased dosing, no symptom development or no administration of bone marrow–stimulating agents.

### Serological evaluation for anti-human antibodies

Two (6.7%) of the brodalumab-treated patients tested positive for anti-brodalumab-binding antibodies, with both positives observed only on day 127. Both binding antibody samples tested negative for the presence of neutralizing anti-brodalumab antibodies. None of the placebo-treated patients tested positive for the presence of anti-brodalumab-binding antibodies.

### Pharmacokinetic analysis

**Figure 1:**
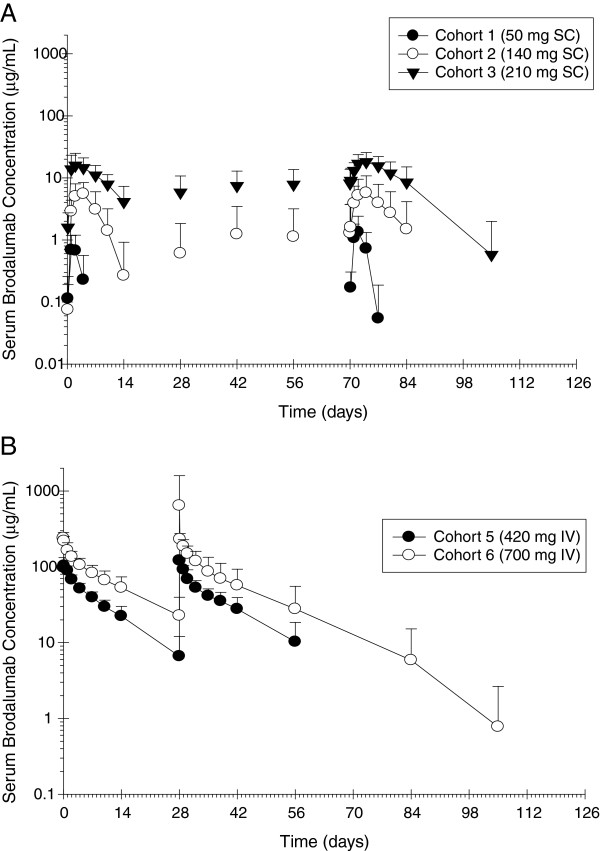
**Mean (SD) serum brodalumab concentration × time profiles (semilog) in rheumatoid arthritis patients following (A) repeat subcutaneous administration and (B) repeat intravenous administration.** IV, intravenous; SC, subcutaneous.

Brodalumab exhibited nonlinear pharmacokinetics in patients with RA. Following a single dose or multiple SC doses of brodalumab across a dose range of 50 to 210 mg, the maximum serum concentration (C_max_) and the area under the serum concentration time curve (AUC_0-*t*_) increased greater than dose proportionally (Table 3 and Figure 1). Accumulation, measured as the ratio of AUC_0-*t*_ following the final dose/AUC_0-*t*_ following the first dose, was less than twofold following multiple-dose SC (140 or 210 mg) or IV (420 or 700 mg) administration of brodalumab (Figure 1). The median time to C_max_ (*t*_max_) was approximately 2 to 4 days following either a single SC dose or six SC doses of brodalumab at 50, 140 or 210 mg brodalumab (Figure 1).

**Table 3:** **Mean pharmacokinetic parameter estimate of brodalumab following multiple subcutaneous and intravenous doses given to rheumatoid arthritis patients**^**a**^

	**Predicted human exposure after multiple SC (*n*** **= 6) or IV (*n*** **= 2) doses**		**Exposure margin based on exposure in monkeys after 3-month weekly SC doses or 1-month weekly IV doses**^**b**^	
**Dose (mg)**	**AUC**_**0-** ***t***_^**c **^**(μg/h/ml)**	**C**_**max **_**(μg/ml)**	**AUC (μg/h/ml)**	**C**_**max **_**(μg/ml)**
50 (SC)	71.3	0.947	4,458	1,246
140 (SC)	2170	9.05	146	130
210 (SC)	6610	23.2	48	51
420 (IV)	24,800	113	126	89
700 (IV)	48,600	198	64	51

^a^AUC, area under the curve; AUC_0-*t*_, area under the serum concentration time curve; C_max_, maximum serum concentration; IV, intravenous; SC, subcutaneous. ^b^No adverse effect level for monkeys is 90 mg/kg SC or 350 mg/kg IV. AUC_0-168 hours_ and C_max_ after the 12th dose of 90 mg/kg weekly SC were 159,000 μg/h/ml and 1,180 μg/ml, respectively. AUC_0-168 hours_ and C_max_ after fourth dose of 350 mg/kg weekly IV were 782 000 μg/h/ml and 10,100 μg/ml, respectively. ^c^AUC_0-*t*_ = AUC_0-336 hours_ for SC cohorts and AUC_0-672 hours_ for IV cohorts.

### Pharmacodynamic measures following administration of brodalumab

**Figure 2:**
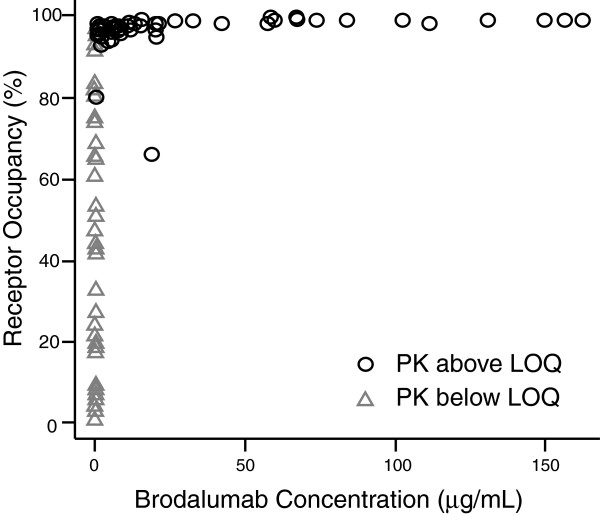
**Relationship between interleukin 17 receptor occupancy levels and serum brodalumab concentrations.** Each symbol represents an individual sample collected during the study for all patients receiving brodalumab. Circles indicate samples with detectable pharmacokinetic (PK) levels of serum brodalumab, and triangles indicate samples below the level of quantitation (LOQ).

Mean IL-17 RO on circulating leukocytes in whole blood from placebo patients was minimal (less than 3%) at all time points. Treatment with brodalumab resulted in dose-dependent increases in IL-17 RO on circulating leukocytes, with mean IL-17 RO increases greater than 95% on day 3 for all dose levels (data not shown). The mean (SD) predose trough levels on days 15, 43 and 85 in the 50 mg, 140 mg and 210 mg brodalumab groups were 34.6% (4.0), 91.3% (2.9) and 97.2% (0.2), respectively. IL-17 RO in the brodalumab IV groups (420 mg and 700 mg) was 99% on day 3 and predose trough levels were generally above 90% on day 29 for both IV cohorts. RO was nearly full at any time point when the drug was measurable. When brodalumab concentrations were above the lower limit of quantification, occupancy 92% or higher was observed at all but two time points (Figure 2). At one time point, the brodalumab serum concentration was very low (0.056 μg/ml). These data demonstrate the limits of the ability to measure unbound (that is, free) brodalumab in circulation, given the high levels of target on circulating cells and the high affinity of brodalumab for IL-17RA. These data demonstrate effective binding of the antibody to target throughout the periods of measured exposure.

To investigate a functional biological effect of brodalumab on modulating baseline IL-17R signaling, an *ex vivo* assay measured changes in the expression of IL-6 mRNA pre- and postdose with brodalumab in the presence of increasing levels of exogenously added IL-17. In patients with a strong IL-6 mRNA response, competitive inhibition of IL-17R signaling caused an increase in EC_50_ (concentration of IL-17 causing a 50% increase in IL-6 mRNA) after brodalumab administration, with minimal changes in magnitude of the IL-6 response. However, the magnitude of the IL-6 response was attenuated in many RA patients before brodalumab administration, with a lower magnitude of IL-6 mRNA production compared to healthy volunteers or psoriasis patients [16]. As a result, accurate measurements of changes in EC_50_ was not possible in all patients, and pre- and postdose samples from RA patients were investigated for potential normalization of reduced magnitude of IL-6 response on days 15 and 43 (when IL-17 RO was high). While predose samples from several patients displayed the attenuated IL-6 mRNA response, no significant increase in the magnitude of the IL-6 mRNA response was observed following treatment with brodalumab compared to placebo (data not shown).

### Clinical responses to brodalumab

On day 85 (week 13), ACR20 had been achieved by 11 (37%) of 30 of patients receiving brodalumab and 2 (22%) of 9 of patients receiving placebo (Table 4). ACR50 scores were observed in one of nine and two of thirty placebo and brodalumab patients, respectively, on day 85. At day 85 (week 13), mean (SD) DAS28 scores were 4.76 (1.41) and 4.39 (1.13) in the brodalumab and placebo groups, respectively. These data represent modest decreases from baseline values of 5.58 (1.26) and 5.54 (1.20), respectively, with a maximum mean change throughout the study of −0.81 (0.86) at day 85 (week 13) for brodalumab patients and −1.34 (0.90) at day 57 for placebo patients (data not shown).

**Table 4:** **ACR20/50/70 responses for patients receiving brodalumab or placebo**^**a**^

					**Brodalumab**						
		**Placebo**			**SC**			**IV**			
**Response, *n*/*N *** **(%)**		**SC**	**IV**	**All**	**50 mg**	**140 mg**	**210 mg**	**420 mg**	**700 mg**	**All**	**Total**
		**(*N*** **= 6)**	**(*N*** **= 4)**	**(*N*** **= 10)**	**(*N*** **= 6)**	**(*N*** **= 6)**	**(*N*** **= 6)**	**(*N*** **= 6)**	**(*N*** **= 6)**	**(*N*** **= 30)**	**(*N*** **= 40)**
ACR20	Day15	2/5 (40)	1/4 (25)	3/9 (33)	0/5 (0)	0/6 (0)	1/6 (17)	3/6 (50)	3/6 (50)	7/29 (24)	10/38 (26)
	Day 29	1/4 (25)	2/4 (50)	3/8 (38)	2/6 (33)	2/6 (33)	0/6 (0)	3/6 (50)	2/6 (33)	9/30 (30)	12/38 (32)
	Day 57	3/5 (60)	2/4 (50)	5/9 (56)	1/6 (17)	0/6 (0)	1/6 (17)	3/6 (50)	4/5 (80)	9/29 (31)	14/38 (37)
	Day 85	2/6 (33)	0/3 (0)	2/9 (22)	2/6 (33)	2/6 (33)	1/6 (17)	2/6 (33)	4/6 (67)	11/30 (37)	13/39 (33)
ACR50	Day15	0/6 (0)	0/4 (0)	0/10 (0)	0/5 (0)	0/6 (0)	0/6 (0)	0/6 (0)	1/6 (17)	1/29 (3)	1/39 (3)
	Day 29	1/5 (20)	0/4 (0)	1/9 (11)	0/6 (0)	0/6 (0)	0/6 (0)	1/6 (17)	0/6 (0)	1/29 (3)	2/39 (5)
	Day 57	1/6 (17)	0/4 (0)	1/10 (10)	0/6 (0)	0/6 (0)	0/6 (0)	0/6 (0)	0/6 (0)	0/30 (0)	1/40 (3)
	Day 85	1/6 (17)	0/3 (0)	1/9 (11)	1/6 (17)	1/6 (17)	0/6 (0)	0/6 (0)	0/6 (0)	2/30 (7)	3/39 (8)
ACR70	Day15	0/6 (0)	0/4 (0)	0/10 (0)	0/5 (0)	0/6 (0)	0/6 (0)	0/6 (0)	0/6 (0)	0/29 (0)	0/39 (0)
	Day 29	0/6 (0)	0/4 (0)	0/10 (0)	0/6 (0)	0/6 (0)	0/6 (0)	0/6 (0)	0/6 (0)	0/30 (0)	0/40 (0)
	Day 57	1/6 (17)	0/4 (0)	1/10 (10)	0/6 (0)	0/6 (0)	0/6 (0)	0/6 (0)	0/6 (0)	0/30 (0)	1/40 (3)
	Day 85	0/6 (0)	0/3 (0)	0/9 (0)	0/6 (0)	0/6 (0)	0/6 (0)	0/6 (0)	0/6 (0)	0/30 (0)	0/39 (0)

^a^ACR20, 20% American College of Rheumatology improvement criteria; ACR50, 50% American College of Rheumatology improvement criteria; ACR70, 70% American College of Rheumatology improvement criteria; IV intravenous, SC subcutaneous. All data are mean (SD) unless otherwise indicated.

## Discussion

IL-17 is an innate proinflammatory cytokine that has been shown to contribute to psoriasis pathogenesis [7] and a subset of additional inflammatory diseases, including RA and PsA [13,14] but not Crohn’s disease [17]. Signaling through the IL-17R complex (IL-17RA:IL-17RC for IL-17A, IL-17 F and IL-17A/F) by various IL-17 ligands promotes host defense through effects on neutrophil homeostasis and the elaboration of proinflammatory factors, including cytokines, chemokines and antimicrobial peptides. The primary goal of this study was to assess the safety, tolerability, pharmacokinetics and immunogenicity of multiple doses of brodalumab in patients with RA. The planned dose expansion portion of this study, which included a primary efficacy endpoint in RA, was not performed. Instead, a separate phase II multiple-dose study was conducted to evaluate the efficacy of brodalumab in patients with RA.

Analysis of the safety data showed an acceptable safety profile for brodalumab. Three SAEs, none of which were considered related to the investigational product, were reported in two patients. One patient (placebo IV) was discontinued from the study early because of an RA flare. There was no evidence of a significant increase in infectious AEs in patients receiving brodalumab. No deaths, dose-limiting toxicities or neutralizing anti-brodalumab antibodies were reported. The incidence of treatment-emergent AEs was similar between the brodalumab and placebo groups. No clinically significant abnormalities in electrocardiograms, safety laboratory values or vital signs were reported. These findings are consistent with the overall safety profile of brodalumab in larger studies of patients with psoriasis [7].

Brodalumab exhibited nonlinear pharmacokinetics in patients with RA after SC administration within a dose range of 50 to 210 mg. Serum brodalumab exposure, as measured by C_max_ and AUC_0-*t*_, increased more than dose-proportionally across the dose range of 50 to 210 mg after single or multiple SC doses of brodalumab given to RA patients. Treatment with brodalumab resulted in dose-dependent increases in IL-17 RO on circulating leukocytes and clear evidence of prolongation of the duration of greater than 90% RO by brodalumab. RO greater than 90% in the presence of any measurable brodalumab, along with numerous RO measurements demonstrating significant target coverage in the absence of measurable brodalumab, indicate that some IL-17R coverage on circulating leukocytes is sustained following the disappearance of measurable concentrations of brodalumab. However, the functional effect of this low-level coverage is unknown and may be minimal. Moderate concentrations of IL-17 ligand would be expected to be able to displace brodalumab at these levels.

Exploratory efficacy analyses, including widely used composite indices of signs and symptoms of RA (ACR20 and DAS28), did not reveal any clinical responses to brodalumab in patients with RA. Twelve weeks after study initiation, 15% of patients receiving brodalumab and 7.5% of patients given placebo achieved ACR50 responses. The single ACR70 response in this study was observed in a placebo patient. Individual ACR components also failed to show a difference between placebo- and brodalumab-treated patients. This small efficacy data set does not provide confidence that inhibition of IL-17R with brodalumab at doses up to 210 mg SC every other week or 700 mg IV monthly is likely to have significant impact on short-term clinical outcomes in RA. The mechanism of this nonresponse and differences from clinical responses observed in other diseases is unknown. Potential explanations for the lack of response include insufficient power to detect modest improvements in this small study and mechanisms that may be specific to blockade of IL-17RA. Receptor blockade could affect multiple ligands (including IL-25) with unknown functions in RA or may require higher levels of target coverage in the synovium. Notably brodalumab has shown evidence of a clinical effect in psoriasis following single and multiple administrations at the same dose level as that used in this RA study [7,18]. Clinical studies powered to assess the clinical efficacy of brodalumab in both MTX-resistant RA and PsA have been completed (Trial registrations: Clinicaltrials.gov NCT00950989 and NCT01516957) [19]. These phase II data will enable a more thorough comparison of the efficacy response observed with brodalumab with therapeutics targeting IL-17A, particularly in RA cases where responses have been mixed and equivocal [20,21].

## Conclusion

In this small, ascending-dose, phase Ib study, we have demonstrated that multiple SC and IV doses of brodalumab were tolerated by patients with active RA. Conclusions about the efficacy of brodalumab in RA patients cannot be reached on the basis of these results, given the study design, although limited clinical effect and no clear dose–response relationship was observed in RA patients receiving brodalumab.

## Abbreviations

ACR: American College of Rheumatology; AE: Adverse event; AUC: Area under the curve; Cmax: Maximum serum concentration; DAS28: Disease activity score in 28 joints; DMARD: Disease-modifying antirheumatic drug; ELISA: Enzyme-linked immunosorbent assay; IL-17: Interleukin 17; IL-17RA: Interleukin 17 receptor type A; LLOQ: Lower limit of quantification; RA: Rheumatoid arthritis; RO: Receptor occupancy; tmax: Median time to C_max_; TNF: Tumor necrosis factor.

## Competing interests

DAM, DS, ES, CBT and JBC are employees and stockholders of Amgen Inc. HD was an employee and stockholder of Amgen Inc at the time of this study.

## Authors’ contributions

DAM, MC, LFFS, MHC, DW, RM, KP and JLK participated in data collection and study coordination. JBC participated in the design of the study. ES, DS and CBR carried out the bioassay and data analysis. HD participated in data management and biostatistical analysis. All authors helped draft the manuscript and participated in manuscript revision. All authors read and approved the final manuscript.

## Participating institutions

Wallace Rheumatic Study Center, Los Angeles, CA; Sarasota Arthritis Research Center, Sarasota, FL; West Michigan Rheumatology, PLLC, Grand Rapids, MI; Physician Research Collaboration, Lincoln, NE; Scleroderma Program, Domino’s Farm, Ann Arbor, MI; Southern Arizona Rheumatology Associates, Tucson, AZ; Dedicated Phase 1, Phoenix, AZ; The Arthritis Program Research Group Inc, Newmarket, ON, Canada; Manitoba Clinic, Winnipeg, MB, Canada; Pharmacological and Biotechnological Center, Médica Sur, Mexico City, Mexico; and Clinical Research Center of Morelia, Michoacan, Mexico.
